# Sleep macro‐architecture, nocturnal hypoxemia, and Alzheimer's disease‐related MRI patterns among diverse older adults

**DOI:** 10.1002/alz.70280

**Published:** 2025-05-19

**Authors:** Dillys Xiaodi Liu, Meredith N. Braskie, Clémence Cavaillès, Carrie Peltz, Susan Redline, Kristine Yaffe

**Affiliations:** ^1^ Department of Psychiatry and Behavioral Sciences University of California San Francisco California USA; ^2^ Mark and Mary Stevens Neuroimaging and Informatics Institute, Keck School of Medicine University of Southern California Los Angeles California USA; ^3^ Northern California Institute for Research and Education San Francisco California USA; ^4^ Division of Sleep Medicine and Circadian Disorders, Department of Medicine Brigham and Women's Hospital and Harvard Medical School Boston Massachusetts USA; ^5^ Department of Psychiatry, Neurology, and Epidemiology and Biostatistics University of California San Francisco California USA

**Keywords:** Alzheimer's disease, dementia, magnetic resonance imaging, sleep, sleep apnea

## Abstract

**INTRODUCTION:**

Sleep patterns change with aging, yet their relationship with brain health, particularly Alzheimer's disease (AD)‐related MRI patterns among diverse older adults is not clear.

**METHODS:**

We cross‐sectionally studied community‐dwelling Non‐Hispanic White (NHW), Hispanic, and Black participants recruited from the Health and Aging Brain Study‐Health Disparities (HABS‐HD)‐Dormir Study, who underwent WatchPAT and brain MRI.

**RESULTS:**

A total of 842 participants (34% male; 42% NHW, 33% Hispanic, and 25% Black; mean age 66.1 ± 8.6 years) were included. Greater light sleep percentage and longer rapid‐eye movement (REM) sleep latency were independently associated with thinner cortex in AD‐signature regions, while inverse pattern was observed for greater deep sleep percentage. Higher Apnea‐Hypopnea Index (AHI) in REM sleep was independently associated with greater white matter hyperintensities volume. There were no ethnoracial interactions for these associations.

**DISCUSSION:**

Light and deep sleep percentage, REM sleep latency, and AHI in REM sleep were associated with AD‐related MRI patterns.

**Highlights:**

- Greater light sleep percentage and longer rapid‐eye movement sleep latency were associated with thinner cortex in the Alzheimer's Disease‐siganture regions, while greater deep sleep percentage was associated with thicker cortex in those regions.
- Higher apnea hypoonea index in rapid eye movement sleep was associated with greater white matter hyperintensities volume.
- No moderation effects by race/ethnicity were observed among Non‐Hispanic White/Hispanic/Black adults.

## 1 BACKGROUND

Sleep patterns change with aging, including disruptions in sleep macro‐architecture^1^ and an increased rate of sleep apnea.^2^ Both disruptions in sleep macro‐architecture (such as reduced slow‐wave sleep) and nocturnal hypoxemia [indicated by elevated Apnea‐Hypopnea Index (AHI) and decreased oxygen saturation] have been linked to increased risk of Alzheimer's disease (AD) and related dementias (AD/ADRD) or magnetic resonance imaging (MRI) markers of brain aging,^3^, ^4^, ^5^, ^6^ suggesting they potentially be the modifiable risk factors for AD/ADRD.^7^, ^8^ Moreover, sleep patterns may vary across racial/ethnic groups,^9^ recent studies analyzing self‐reported sleep data from the National Health Interview Survey indicate that Hispanics are more likely to have a shorter sleep duration compared to Non‐Hispanic White (NHW) group. Previous findings from the Dormir study also reported higher prevalence of rapid eye movement (REM) sleep apnea among Black adults compared to NHW adults.^10^ However, the relationships between sleep macro‐architecture, nocturnal hypoxemia, and AD‐related MRI patterns—and whether they are consistent across NHW, Hispanic, and Black groups—remain unclear.

AD‐related MRI patterns including AD‐signature cortical thickness, white matter hyperintensities (WMH), and hippocampal volume, are the core pathological markers of neurodegeneration (N) in the 2018 AT(N) framework.^11^ Specifically, cortical thinning in certain regions of the temporal lobe is recognized as the cortical signature of AD‐related neurodegeneration, and could be a useful biomarker for predicting AD/ADRD in cognitively normal adults.^12^ There are limited data that assess objective sleep parameters and AD‐related MRI patterns, often involving small sample sizes or a lack of consideration for racial/ethnic diversity.^4^, ^6^ Understanding the relationship between sleep macro‐architecture, nocturnal hypoxemia and AD‐related MRI patterns—and whether these associations are moderated by racial/ethnic diversity, could provide insights for identifying modifiable risk factors and reducing health disparities in AD/ADRD.

This study aimed to determine whether objectively‐measured sleep metrics, including sleep macro‐architecture and nocturnal hypoxemia, are independently associated with AD‐related MRI biomarkers in a cohort of race/ethnicity diverse individuals across the cognitive spectrum. We hypothesized that greater light sleep percentage, longer REM sleep latency, smaller deep or REM sleep percentage, and greater nocturnal hypoxemia would be associated with worse AD‐related MRI patterns, with race/ethnicity as a potential moderator.

## 2 METHODS

### 2.1 Participants and study design

The Dormir Study is an ancillary study of the Health and Aging Brain Study‐Health Disparities (HABS‐HD) study, conducted from 2020 to 2024. It aims to examine the link between objectively measured sleep and brain aging in a racially and ethnically diverse population.^10^, ^13^ In brief, HABS‐HD is an ongoing, longitudinal community‐based multi‐ethnic study recruiting NHW, Hispanic, and Black elders (50 years and above). HABS‐HD participants were further invited to join the Dormir Study for a comprehensive sleep assessment, including an United States Food and Drug Administration (FDA)‐approved Type 3 home sleep test (WatchPAT), 7‐day actigraphy, and a sleep diary. Individuals with permanent pacemakers (atrial pacing or VVI without sinus rhythm) and those currently using a continuous positive airway pressure machine with oxygen were excluded from the Dormir study, as the WatchPAT device is not indicated for use in these populations. Here, we focus on participants with evaluable overnight WatchPAT and brain MRI data on the baseline visit. Of the 996 Dormir participants, 50 did not have evaluable brain MRI and 104 did not have evaluable WatchPAT data, resulting in our analytic cohort of 842 participants.

**RESEARCH IN CONTEXT**

1. **Systematic review**: The authors reviewed literature on sleep and Alzheimer's disease (AD)‐related magnetic resonance imaging (MRI) patterns using PubMed. Increasing evidence has linked poor sleep to poorer brain health. However, most prior studies based their results on self‐reported sleep measures, or used nonspecific morphometric imaging markers, or did not account for racial/ethnic diversity.
2. **Interpretation**: Using WatchPAT‐extracted sleep metrics and AD‐related MRI markers from the recently completed Health and Aging Brain Study‐Health Disparities (HABS‐HD)‐Dormir Study, we found that greater light sleep percentage and longer rapid‐eye movement (REM) sleep latency were associated with thinner cortex in AD‐signature regions, while greater deep sleep percentage was associated with thicker cortex in those regions. Higher Apnea‐Hypopnea Index in REM sleep was associated with greater white matter hyperintensities volume.
3. **Future directions**: Future studies are warranted to evaluate the long‐term pattern of these associations and assess whether treatments for poor sleep and nocturnal hypoxemia will be helpful to brain health.

### 2.2 WatchPAT methods

We performed overnight WatchPAT (WP200U, Itamar Medical Ltd, Israel) for each Dormir participant after clinical evaluation. Sleep records were first processed using Itamar's zzzPAT software (versions 5.2.79.7p and 4.6.71.7, according to the calendar dates of the study) by trained scorers at the Brigham and Women's Hospital Sleep Reading Center. Records were then manually annotated following the criteria published previously.^14^ In brief, respiratory events in non‐REM sleep were retained if they showed a heart rate increase, a peripheral arterial tone (PAT) decrease, and ≥ 3 percent desaturation, with no postural change. Respiratory events in REM sleep were retained when associated with a ≥ 4% desaturation. REM and wake periods were also annotated based on visual review using the templates published previously.^14^ Compared to polysomnography, this approach was reported to yield accurate estimates of the AHI (total number of apnea and hypopnea events divided by the valid sleep time; r = 0.81), total sleep time (r = 0.73) and REM duration (r = 0.64). Moderate accuracy for estimating duration of deep and light sleep have been reported for the zzzPAT algorithm.^15^ The key sleep metrics studied in this analysis were percentages of light, deep, or REM sleep, REM sleep latency, overall AHI, AHI in REM or non‐REM sleep, and mean oxygen saturation. All sleep metrics, except for mean oxygen saturation, were standardized into Z‐score for further analysis. The mean oxygen saturation was calculated based on oxygen level across total valid sleep time and categorized as < 94% versus ≥ 94% (the median value of the study sample).^16^

### 2.3 MRI data acquisition and analysis

All participants underwent a 3 Tesla (T) brain MRI either on a Siemens MAGNETOM Skyra whole‐body scanner, or a Siemens MAGNETOM Vida scanner (Siemens Healthineers AG; Erlangen, Germany) within 1 month after clinical evaluation, according to a standard protocol as described previously.^17^ All the images were reviewed by a trained neuroradiologist.

AD‐signature cortical thickness, hippocampal volume, and intracranial volume were derived from T1‐weighted Magnetization Prepared RApid Gradient Echo (MPRAGE) sequence (Skyra: TR = 2300 ms; TE = 2.93 ms; 1.1 x. 1.1 × 1.2 mm; Vida: TR = 2300 ms; TE = 2.98 ms; 1.0 x. 1.0 × 1.0 mm) via FreeSurfer 5.3.0.^18^, ^19^ The standardized AD‐signature cortical thickness is a well‐established AD biomarker, calculated based on the surface‐area weighted mean cortical thickness across individual regions of interests, including the entorhinal cortex, fusiform gyrus, inferior temporal gyrus, and middle temporal gyrus.^20^ We used HippoDeep software to segment the right and left hippocampus and to estimate hippocampal and intracranial volume (ICV). Hippocampal volume was evaluated as the mean of left and right hippocampal volume. WMH volume was processed and segmented based on T1‐MPRAGE and fluid‐attenuated inversion recovery (FLAIR) sequences using the Lesion Segmentation Toolbox Lesion Growth Algorithm in Statistical Parametric Mapping (SPM) software, as published previously.^21^ We removed outliers in WMH volume that were outside 3 standard deviations (SDs) of the mean difference, then log transformed it using log(WMH+1). All the imaging values that did not pass quality control were marked as missing.

We normalized hippocampal volume and log(WMH+1) volume by ICV using the residual approach. Specifically, both volumes were regressed against ICV, and the residuals from the regression model were used as ICV‐adjusted volumes. All the three MRI biomarkers were standardized by calculating Z‐scores using the sample means and SDs. As the normalized log(WMH+1) volume was right‐skewed, we further categorized it into tertiles, with the top tertile representing the largest burden and the lowest tertile the smallest burden (Figure S1).

### 2.4 Covariates

Participants underwent a clinical evaluation to assess demographics and medical history. Race/ethnicity were self‐reported and classified as NHW, Hispanic, or Black. Medical history was determined by a consensus of self‐report and clinical diagnosis. Research cognitive diagnosis (mild cognitive impairment [MCI] or dementia) was determined based on consensus review (self‐report and informant‐report assessments of daily functioning, expert clinician‐assigned Clinical Dementia Rating scores [based on both daily functioning and cognitive performance], and results from neuropsychological testing) as described previously.^13^ Sleep medication, anti‐depressant use, smoking, and alcohol consumption were based on self‐report. Body mass index (BMI) was calculated based on weight and height.

### 2.5 Statistical analysis

We described baseline characteristics stratified by race/ethnicity using mean (SD), median (interquartile range [IQR]) or number (percentage), as appropriate. We imputed BMI (1.2% missing) using the median value.

We evaluated the associations between sleep macro‐architecture, nocturnal hypoxemia, and AD‐signature cortical thickness or hippocampal volume, using three linear regression models: model 1 was unadjusted; model 2 was adjusted for age, sex, and race/ethnicity; model 3 was additionally adjusted for BMI, educational years, cognitive status (normal vs. MCI or dementia), depressive status (history of depression or on anti‐depressant), smoking, alcohol consumption, time interval between WatchPAT and MRI, and MRI scanner. We checked and verified linear regression model assumptions. For the AD‐signature cortical thickness analyses, our sample was *n* = 636, and for the hippocampal volume analyses, a sample of *n* = 777. We assessed the relationship between sleep macro‐architecture, nocturnal hypoxemia, and tertiles of WMH volume using the ordinal logistic model in a similar approach as described above, with *n* = 842. We also conducted a sensitivity analysis that excluding participants with MCI or dementia.

We additionally explored race/ethnicity as a moderator by adding two‐way (primary sleep predictor × race/ethnicity) to each fully adjusted model, respectively. We further repeated all the regression analysis stratified by racial/ethnic groups to evaluate whether there were racial/ethnic‐specific associations.

We reported results using standardized *β* coefficient of linear regression or odds ratio of ordinal logistic regression for all the continuous sleep exposures, with 95% confidence interval (95% CI) and false discovery rate (FDR)‐corrected *p* value. The statistical significance level was set as *p *< 0.05. All analyses were conducted using Python version 3.12.2 (Python Software Foundation) and R version 4.4.1 (R Foundation).

## 3 RESULTS

### 3.1 Sample characteristics

The mean age of the Dormir participants was 66.1 years (SD = 8.6), 34% (*n* = 285) were male, 42% (*n* = 352) were NHW, 33% (*n* = 278) were Hispanic, and 25% (*n* = 212) were Black participants. Seventy‐eight percent (*n* = 654) were cognitively normal and 22% had MCI (*n* = 158) or dementia (*n* = 30). The median time interval between WatchPAT and brain MRI was 0.6 year (IQR = 0.5). Compared to NHW participants, Hispanic and Black participants tended to be younger and more often female, had higher BMI and fewer years of education, were more likely to have hypertension, diabetes, and MCI/dementia, and less likely to take anti‐depressant, sleep medication, or alcohol (all *p* < 0.05) (Table 1).

**TABLE 1:** Baseline characteristics of the study sample.

Baseline characteristics, mean (SD), median [IQR], or *N* (%)	NHW participants *N* = 352	Hispanic participants *N* = 278	Black participants *N* = 212	*p‐*value
**Demographic**				
Age, year	69.8 (8.1)	64.0 (7.9)	62.8 (7.9)	<0.001
Male	142 (40)	76 (27)	67 (32)	0.002
Education, year	16 (3)	11 (4)	15 (3)	<0.001
Body mass index	29.7 (6.5)	30.8 (6.1)	32.9 (6.8)	<0.001
Ever/current smoker	312 (37)	133 (38)	104 (37)	0.84
Alcohol consumption				<0.001
Never drink	116 (33)	157 (56)	83 (39)	
1–4 times per month	117 (33)	99 (36)	96 (45)	
2–3 times per week	50 (14)	14 (5.0)	22 (10)	
≥4 times per week	69 (20)	8 (2.9)	11 (5.2)	
**Medical history**				
Hypertension	208 (59)	167 (60)	170 (80)	<0.001
Hyperlipidemia	250 (71)	207 (74)	137 (65)	0.06
Diabetes	53 (15)	93 (33)	65 (31)	<0.001
Cerebrovascular disease	26 (7.4)	13 (4.7)	19 (9.0)	0.2
MCI or dementia	51 (14)	72 (26)	65 (31)	<0.001
Depression	296 (35)	123 (35)	111 (40)	0.05
**Medications**				
Anti‐depressant	67 (19)	31 (11)	32 (15)	0.02
Sleep medication	68 (19)	30 (11)	32 (15)	0.01
**Sleep macro‐architecture**				
Total sleep time, hour	7.0 (1.2)	6.8 (1.3)	6.6 (1.3)	<0.001
Light sleep percentage	63.1 (10.5)	59.0 (10.5)	59.1 (11.3)	<0.001
Deep sleep percentage	14.4 (5.7)	16.9 (6.0)	17.6 (6.8)	<0.001
REM sleep latency, hour	1.6 (1.1)	1.6 (1.1)	1.5 (1.0)	0.33
REM sleep percentage	22.5 (7.0)	24.1 (6.7)	23.3 (7.0)	0.02
**Nocturnal hypoxemia**				
AHI	17.3 (12.3)	19.0 (14.0)	20.4 (15.4)	0.06
AHI in non‐REM sleep	15.0 (12.4)	16.0 (14.1)	16.8 (15.6)	0.60
AHI in REM sleep	24.6 (15.0)	28.1 (16.4)	31.2 (17.6)	<0.001
Mean oxygen saturation, %	93.5 (1.6)	94.2 (1.6)	93.3 (1.5)	<0.001
**Neuroimaging**				
AD‐signature cortical thickness, mm	2.77 (0.13)	2.76 (0.13)	2.78 (0.12)	0.52
(Missing)	90	49	67	
WMH volume, mL	0 [1.03]	0 [0.38]	0.54 [1.73]	<0.001
Hippocampal volume, mm^3^	3,219 [500]	3,202 [441]	3,198 [485]	0.51
(Missing)	27	17	21	
Intracranial volume, mL	1,439 [209]	1,354 [164]	1,392 [167]	<0.001
Interval between WatchPAT and brain MRI, year	0.6 [0.5]	0.5 [0.4]	0.7 [0.4]	<0.001

Abbreviations: AHI, Apnea‐Hypopnea Index; AD, Alzheimer's disease; IQR, interquartile range; MCI, mild cognitive impairment; MRI, magnetic resonance imaging; NHW, Non‐Hispanic White; REM, rapid eye movement; SD, standard deviation; WMH, white matter hyperintensities.

Moreover, compared to NHW, Hispanic and Black participants tended to have shorter total sleep time, less light sleep percentage, greater deep and REM sleep percentage, and higher AHI in REM sleep; Hispanic participants tended to have higher mean oxygen saturation, while Black participants tended to have lower mean oxygen saturation (all *p <* 0.05). No significant difference was found in REM sleep latency, overall AHI, or AHI in non‐REM sleep among ethnoracial groups (all *p* > 0.05). Black participants had greater WMH volume compared to NHW and Hispanic participants (*p* < 0.05); otherwise, there was no significant difference in AD‐signature cortical thickness or hippocampal volume among ethnoracial groups (all *p* > 0.05) (Table 1).

### 3.2 Associations between sleep metrics and AD‐signature cortical thickness

**FIGURE 1:**
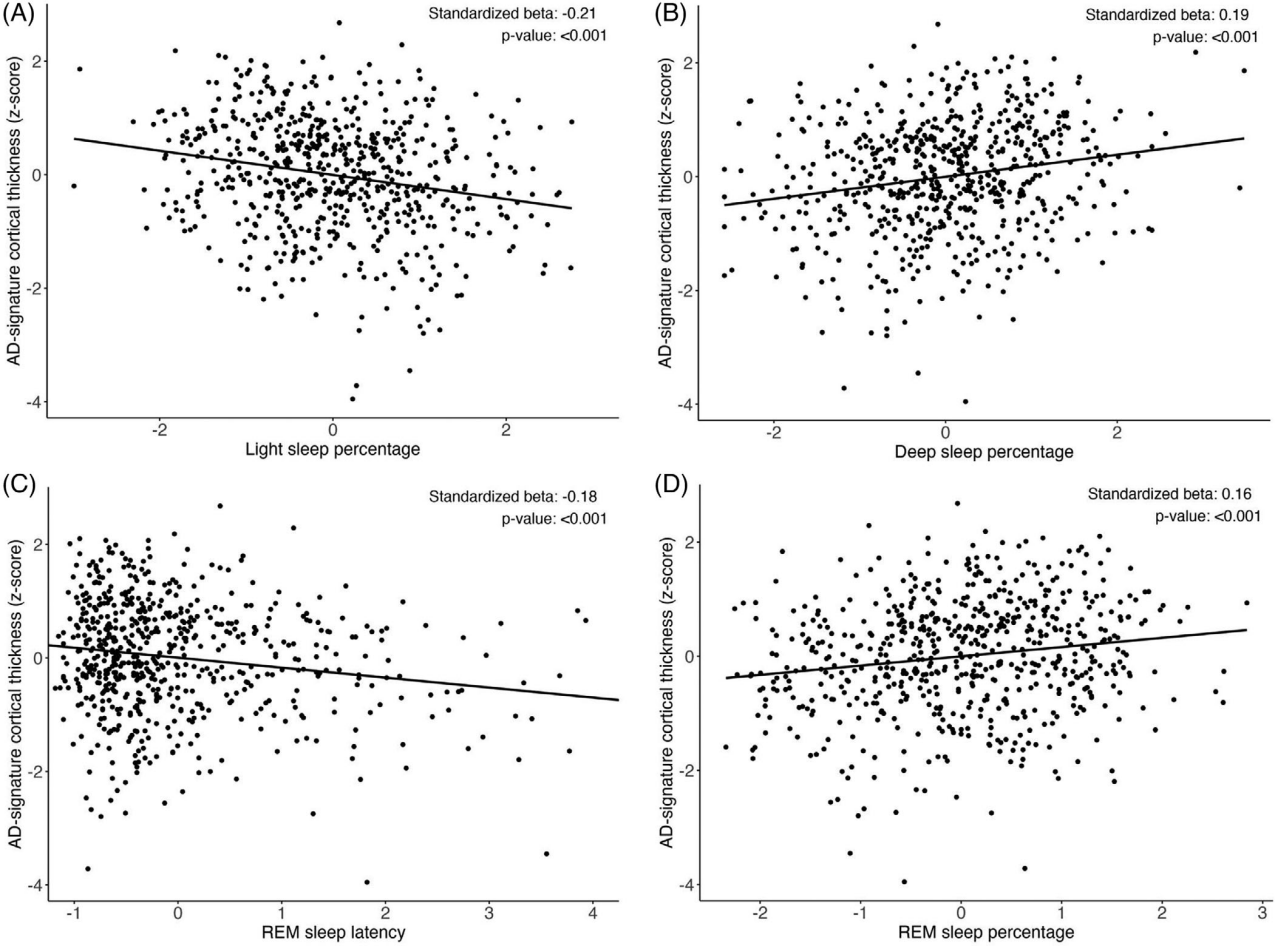
Scatterplot shows unadjusted associations between sleep macro‐architecture and Alzheimer's Disease‐signature cortical thickness. AD, Alzheimer's disease; REM, rapid eye movement.

In unadjusted models, greater light sleep percentage and longer REM sleep latency were associated with thinner cortex in the AD‐signature region, while an inverse pattern was observed for deep and REM sleep percentage in the overall sample and within each ethnoracial group (Figure 1, Table 2). After multivariate adjustment, results remained significant in the overall sample for light and deep sleep percentage and REM sleep latency, but not for REM sleep percentage, with similar trends across three racial/ethnic groups (Table 2, Model 3): *β*_light sleep percentage_ per 1‐SD increase = –0.12, (95% CI –0.19 to –0.05, *p* = 0.009); *β*_deep sleep percentage_ per 1‐SD increase = 0.12 (95% CI 0.05 to 0.20, *p* = 0.007); *β*_REM sleep latency_ per 1‐SD increase = –0.14 (95% CI –0.21 to –0.07, *p <* 0.001); *β*_REM sleep percentage_ per 1‐SD increase = 0.08 (95% CI 0.002 to 0.15, *p *= 0.17). Although no ethnoracial interactions were observed (all interaction *p >* 0.05, Table S1), the associations between REM sleep latency and AD‐signature cortical thickness were stronger in NHW and Hispanic participants compared to Black participants (Table 2). Moreover, higher overall AHI and AHI in non‐REM sleep were associated with thinner cortex in the AD‐signature region in unadjusted models, but these associations were attenuated after multivariate adjustment (all *p >* 0.05, Table S2), with no ethnoracial interactions observed (all interaction *p >* 0.05, Table S1).

**TABLE 2:** Linear regression models investigating associations between sleep macro‐architecture and Alzheimer's Disease‐signature cortical thickness.

	Overall (*n* = 636)		NHW (*n* = 262)		Hispanic (*n* = 229)		Black (*n* = 145)	
	***β* (95% CI)**	***p*‐value**	***β* (95% CI)**	***p*‐value**	***β* (95% CI)**	***p*‐value**	***β* (95% CI)**	***p*‐value**
**Light sleep percentage**								
Model 1	−0.21 (–0.29 to –0.14)	<0.001	−0.16 (–0.29 to –0.03)	0.02	−0.28 (–0.41 to –0.15)	<0.001	−0.22 (–0.36 to –0.07)	0.004
Model 2	−0.12 (–0.19 to –0.04)	0.003	−0.08 (–0.20 to 0.04)	0.27	−0.17 (–0.30 to –0.04)	0.02	−0.10 (–0.24 to 0.04)	0.15
Model 3	−0.12 (–0.19 to –0.05)	0.009	−0.09 (–0.21 to 0.03)	0.42	−0.16 (–0.29 to –0.03)	0.10	−0.12 (–0.26 to 0.03)	0.40
**Deep sleep percentage**								
Model 1	0.19 (0.12 to 0.27)	<0.001	0.12 (–0.02 to 0.26)	0.10	0.26 (0.13 to 0.39)	<0.001	0.21 (0.07 to 0.35)	0.003
Model 2	0.12 (0.04 to 0.19)	0.005	0.06 (–0.07 to 0.19)	0.39	0.16 (0.04 to 0.29)	0.02	0.12 (–0.01 to 0.25)	0.08
Model 3	0.12 (0.05 to 0.20)	0.007	0.08 (–0.05 to 0.21)	0.67	0.17 (0.04 to 0.30)	0.06	0.14 (0 to 0.27)	0.28
**REM sleep latency**								
Model 1	−0.18 (–0.25 to –0.10)	<0.001	−0.17 (–0.29 to –0.05)	0.006	−0.24 (–0.36 to –0.12)	<0.001	−0.09 (–0.25 to 0.08)	0.31
Model 2	−0.15 (–0.22 to –0.08)	<0.001	−0.17 (–0.28 to –0.06)	0.004	−0.18 (–0.29 to –0.06)	0.003	−0.08 (–0.23 to 0.06)	0.27
Model 3	−0.14 (–0.21 to –0.07)	<0.001	−0.16 (–0.26 to –0.05)	0.03	−0.16 (–0.28 to –0.04)	0.05	−0.07 (–0.23 to 0.08)	0.54
**REM sleep percentage**								
Model 1	0.16 (0.09 to 0.24)	<0.001	0.13 (0.01 to 0.26)	0.03	0.20 (0.07 to 0.34)	0.003	0.15 (–0.01 to 0.31)	0.06
Model 2	0.08 (0.01 to 0.15)	0.03	0.07 (–0.04 to 0.18)	0.32	0.11 (–0.02 to 0.24)	0.15	0.04 (–0.10 to 0.19)	0.56
Model 3	0.08 (0.002 to 0.15)	0.17	0.07 (–0.04 to 0.18)	0.63	0.09 (–0.04 to 0.22)	0.40	0.04 (–0.11 to 0.20)	0.69

*Note*. Data are presented as standardized *β* per 1‐SD increase (95% confidence interval) with FDR‐adjusted *p*‐value.

Model 1 unadjusted. Model 2: adjust for age, sex, and race/ethnicity (in the model for overall sample). Model 3: model 2 plus further adjustment for body mass index, education, cognitive status, depressive status (history of depression or on anti‐depressant), smoking, alcohol consumption, time interval between WatchPAT and brain MRI, and MRI scanner.

Abbreviations: CI, confidence interval; FDR, false discovery rate; MRI, magnetic resonance imaging; NHW, Non‐Hispanic White; REM, rapid‐eye movement.

### 3.3 Associations between sleep metrics and WMH volume

**FIGURE 2:**
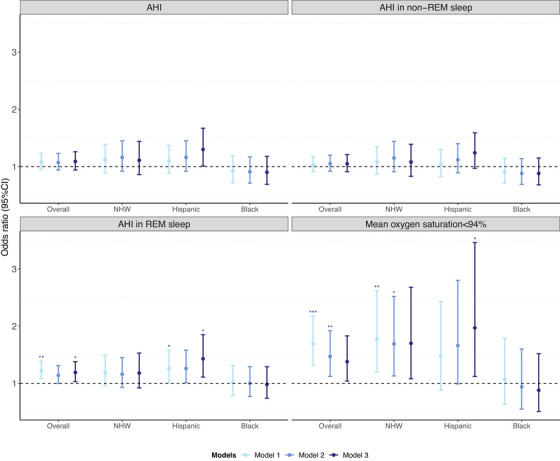
Odds ratios of associations between sleep metrics and WMH volume. WMH volume was categorized into tertiles: the top tertile of WMH (largest WMH burden) to the lowest tertile of WMH (smallest WMH burden), using the lowest tertile as the reference group. Model 1 unadjusted. Model 2: model 1 plus further adjustment for age, sex, and race/ethnicity (in the model for overall sample). Model 3: model 2 plus further adjustment for education, body mass index, cognitive status, depressive status (history of depression or on anti‐depressant), smoking, alcohol consumption, time interval between WatchPAT and brain MRI, and MRI scanner. *FDR‐adjusted *p *< 0.05; **FDR‐adjusted *p *< 0.01; ***FDR‐adjusted *p *< 0.001. AHI, Apnea‐Hypopnea Index; CI, confidence interval; FDR, false discovery rate; MRI, magnetic resonance imaging; REM, rapid‐eye movement; WMH, white matter hyperintensities.

In the overall sample, higher AHI in REM sleep and mean oxygen saturation < 94% were associated with higher WMH tertile in the unadjusted models, these relationships remained significant for AHI in REM sleep, but not for mean oxygen saturation < 94% after multivariate adjustment: OR_AHI in REM sleep_ per 1‐SD increase = 1.19 (95% CI 1.03 to 1.38, *p* = 0.042), OR_Mean oxygen saturation<94%_ = 1.38 (95% CI 1.04 to 1.83, *p *= 0.062); (Figure 2, Model 3). Further adjustment for hypertension yielded similar results. While no significant ethnoracial interactions were detected, the association between AHI in REM sleep, mean oxygen saturation < 94%, and WMH volume was stronger in Hispanic participants compared to NHW and Black participants (Table S3, Figure 2). No significant associations or ethnoracial interactions were found between sleep macro‐architecture, overall AHI, AHI in non‐REM sleep and WMH volume in the overall sample and within each ethnoracial group (Figure 2 & S2, Table S3, all interaction *p >* 0.05).

### 3.4 Associations between sleep metrics and hippocampal volume

In unadjusted models, greater light sleep percentage was associated with smaller hippocampal volume, while greater deep sleep percentage was associated with greater hippocampal volume (Table S4). Similar patterns were observed in the relationship between nocturnal hypoxemia with smaller hippocampal volume, with no significant findings or ethnoracial interaction detected after multivariate adjustment (Table S5 & S6).

### 3.5 Sensitivity analysis

We conducted sensitivity analysis by excluding participants with MCI or dementia. Similar to our main analysis, the significance and magnitude remained consistent among participants with normal cognition.

## 4 DISCUSSION

Among NHW/Hispanic/Black adults, we found that greater light sleep percentage and longer REM sleep latency were significantly associated with thinner cortex in the AD‐signature regions, whereas greater deep sleep percentage was associated with thicker cortex in those regions. In addition, AHI in REM sleep was associated with greater WMH volume. No ethnoracial interactions were detected for these associations.

To the best of our knowledge, our study is the first to report the associations between in‐home, tonometry‐based estimates of light/deep sleep percentage, REM sleep latency, and AD‐signature cortical thickness in a multi‐ethnic population. Our results showed that greater light sleep percentage and longer REM sleep latency are associated with thinner cortex in the AD‐signature regions in diverse populations. In contrast, greater deep sleep percentage is associated with thicker cortex. Prior work showed that facility‐based polysomnography‐based decreased non‐REM sleep stage 1 and greater slow‐wave sleep percentage were associated with higher mean cortical thickness,^22^ but that study did not assess ethnoracial interaction or investigate on cortical thickness in AD‐signature regions, a well‐established MRI biomarker predicting memory decline and the conversion from MCI to dementia over time.^23^, ^24^ Moreover, we found the association of REM sleep latency and AD‐signature cortical thickness were stronger in NHW and Hispanic participants compared to Black participants. Given the lack of significant differences in either REM sleep latency or AD‐signature cortical thickness across the racial/ethnic groups, our results suggest that NHW and Hispanic individuals may be more vulnerable to prolonged REM sleep latency in relation to AD‐signature cortical thinning. Future large‐scale longitudinal studies, particularly those including more Black participants, are warranted to validate these findings, given the smaller representation of Black individuals in our current study compared to NHW and Hispanic participants. The underlying mechanism linking poor sleep and cortical thinning is not certain, dynamic changes in cerebral blood flow may play a role in cortical thinning when sleep macro‐architecture is changed.^25^ The increased light sleep percentage and REM sleep latency observed in our study may be linked to perfusion abnormalities that contribute to AD‐signature cortical thinning, while increased deep sleep percentage helps to maintain cerebral perfusion. Additionally, one study found that oxygen desaturation was related to reduced cortical thickness in temporal lobes among a small sample of older adults at risk for dementia.^3^  Although our cross‐sectional analysis of a large, diverse cohort did not detect any association between nocturnal hypoxemia or REM sleep percentage and AD‐signature cortical thickness, future longitudinal studies are warranted to validate our findings.

Several studies have linked nocturnal hypoxemia to WMH mostly among patients with sleep apnea.^5^, ^6^, ^26^ Our study builds on these works and is the first to report that higher AHI in REM sleep is linked to greater WMH volume. Respiratory events tend to be longer and associated with greater hypoxemia during REM sleep compared to non‐REM sleep. Moreover, sleep apnea during REM is associated with hypertension, cardiovascular and metabolic comorbidities,^27^ and daytime hypoperfusion in the ventromedial prefrontal and fronto‐insular regions,^28^ which may contribute to the pathological pathway leading to WMH formation. We also found that mean oxygen saturation < 94% was numerically associated with greater WMH volume. Low oxygen saturation may trigger neuroinflammation, or hypoperfusion in the brain white matter through recurrent reductions in blood oxygen supply, resulting in WMH progression. Moreover, although we did not detect any ethoracial interactions between nocturnal hypoxemia and WMH volume, the associations of AHI in REM sleep and mean oxygen saturation < 94% with WMH volume were more pronounced in Hispanic participants—whose AHI in REM sleep was intermediate between NHW and Black participants, and who had the highest oxygen saturation—compared to NHW and Black participants. These results indicated that Hispanic individuals might be more susceptible with nocturnal hypoxemia in regard of WMH burden. Future longitudinal studies are needed to explore the relationships between REM sleep apnea and hypoxemia with WMH burden, and to determine whether standard treatments like continuous positive airway pressure can mitigate its negative effects on brain health, particularly in Hispanic populations.

It remains unclear whether sleep metrics affect hippocampal volume. Nocturnal hypoxemia was associated with hippocampal volume in the Rotterdam Study,^29^ but not among patients with sleep apnea.^30^ One study reported that REM hypoxemia was correlated to smaller hippocampal subfield in older adults at risk for dementia,^31^  two other studies did not find any association between self‐reported sleep measures and hippocampal volume across adult lifespan.^32^, ^33^ Similar to those negative results, we did not find any association between sleep macro‐architecture, nocturnal hypoxemia with hippocampal volume after multivariate adjustment. Future longitudinal studies are required to explore whether objectively‐measured sleep metrics are linked to hippocampal volume or its subfields.

Our study has several strengths and limitations. The Dormir study is an ethnically and racially diverse cohort involving representative NHW, Hispanic, and Black adults across cognitive spectrum. We used comprehensive neuroimaging data and objective sleep measures, considering most studies used subjective sleep measures that are not well correlated with sleep stages or sleep apnea severity. We used a validated minimally obtrusive device to measure sleep‐disordered breathing and to estimate sleep stage distributions—a potentially scalable approach that also may provide more representative data than would be obtained using a facility‐based study. We also adjust for several important covariates. We acknowledged several limitations. Given that this is a cross‐sectional study, we cannot determine a causal relationship between sleep metrics and neuroimaging markers. Furthermore, while there are advantages to home‐based sleep assessments, light and deep sleep stage estimation are only moderately accurate, and respiratory event detection did not include direct assessment of flow limitation. In addition, the WatchPAT scoring algorithm applied different criteria to identifying respiratory events in REM versus non‐REM, and further research should confirm the state‐specific findings observed in this study.

In conclusion, our results showed that greater light sleep percentage, longer REM sleep latency, and smaller deep sleep percentage are associated with thinner cortex in AD‐signature regions, while REM‐specific AHI are linked to greater WMH volume, particularly in Hispanic populations. Future studies are warranted to clarify the pathophysiological mechanisms connecting sleep and AD‐related MRI patterns, and whether treatments on poor sleep and nocturnal hypoxemia will be helpful to brain health.

## CONFLICT OF INTEREST STATEMENT

The authors have no conflicts of interests to report Author disclosures are available in the Supporting information

## CONSENT STATEMENT

All study participants provided written informed consent, and the Dormir study was approved by North Texas Regional Institutional Review Board (1516500‐1).

## Supporting information



Supporting Information

Supporting Information
