# Supplementary material for: Sleep macro‐architecture, nocturnal hypoxemia, and Alzheimer's disease‐related MRI patterns among diverse older adults
Source: Alzheimers Dement. 2025 May 19;21(5):e70280. doi: 10.1002/alz.70280 (PMC12089065; doi:10.1002/alz.70280)
Supplement: Supplementary file 2 — Supporting Information [file ALZ-21-e70280-s001.pdf]

Supplementary material

Sleep macro-architecture, nocturnal hypoxemia, and Alzheimer's Disease-related MRI patterns among diverse older adults

List of Supplemental Tables and Figures

Table S1. Interaction *p*-values for sleep metrics × race/ethnicity on Alzheimer's disease-signature cortical thickness

Table S2. Linear regression models investigating associations between nocturnal hypoxemia and Alzheimer's Disease-signature cortical thickness by race/ethnicity

Table S3. Interaction *p*-values for sleep metrics × race/ethnicity on WMH volume

Table S4. Linear regression models investigating associations between sleep macro-architecture and hippocampal volume by race/ethnicity

Table S5. Linear regression models investigating associations between nocturnal hypoxemia and hippocampus volume by race/ethnicity

Table S6. Interaction *p*-values for sleep metrics × race/ethnicity on hippocampal volume

Figure S1. Distribution of WMH volume in tertiles

Figure S2. Odds ratios of the associations between sleep macro-architecture and WMH volume by race/ethnicity

Table S1. Interaction *p*-values for sleep metrics × race/ethnicity on Alzheimer's disease-signature cortical thickness

	**Hispanic**		**Black**	
	β (95% CI)	*p* value for interaction	β (95% CI)	*p* value for interaction
**Sleep architecture**				
Light sleep percentage	−0.07 (−0.24 to 0.10)	0.57	−0.05 (−0.23 to 0.13)	0.68
Deep sleep percentage	0.10 (−0.08 to 0.27)	0.48	0.08 (−0.10 to 0.26)	0.50
REM sleep latency	0.01 (−0.15 to 0.16)	0.95	0.07 (−0.12 to 0.25)	0.70
REM sleep percentage	0.02 (−0.14 to 0.19)	0.96	0.01 (−0.18 to 0.19)	0.99
**Nocturnal hypoxemia**				
AHI	-0.06 (−0.16 to 0.04)	0.94	0.02 (−0.16 to 0.20)	0.92
AHI in non-REM sleep	0.01 (−0.16 to 0.18)	0.97	0 (−0.18 to 0.17)	0.97
AHI in REM sleep	0 (−0.17 to 0.17)	0.97	0.09 (−0.09 to 0.28)	0.49
Mean oxygen saturation<94%	−0.28 (−0.63 to 0.07)	0.37	−0.11 (−0.49 to 0.26)	0.72

Data is presented as standardized β (95% confidence interval) with FDR-adjusted *p* value for interaction
Multivariable model used Non-Hispanic White group as the reference group
AHI, apnea-hypopnea index; CI, confidence interval; FDR, false discovery rate; REM, rapid-eye movement

Table S2. Linear regression models investigating associations between nocturnal hypoxemia and Alzheimer's Disease-signature cortical thickness by race/ethnicity

	Overall (n = 636)		NHW (n = 262)		Hispanic (n = 229)		Black (n = 145)	
	β (95% CI)	*p* value	β (95% CI)	*p* value	β (95% CI)	*p* value	β (95% CI)	*p* value
**AHI**								
Model 1	−0.10 (−0.18 to −0.03)	0.009	−0.13 (−0.27 to 0.01)	0.08	−0.12 (−0.25 to 0)	0.05	−0.06 (−0.20 to 0.07)	0.37
Model 2	−0.08 (−0.15 to −0.01)	0.04	−0.09 (−0.22 to 0.04)	0.27	−0.08 (−0.19 to 0.04)	0.19	−0.06 (−0.19 to 0.06)	0.30
Model 3	−0.08 (−0.15 to 0)	0.17	−0.09 (−0.23 to 0.04)	0.54	−0.07 (−0.20 to 0.05)	0.55	−0.05 (−0.18 to 0.08)	0.70
**AHI in non-REM sleep**								
Model 1	−0.12 (−0.20 to −0.05)	0.002	−0.15 (−0.29 to 0)	0.04	−0.14 (−0.26 to −0.01)	0.03	−0.09 (−0.22 to 0.04)	0.18
Model 2	−0.09 (−0.16 to −0.01)	0.03	−0.09 (−0.22 to 0.05)	0.29	−0.08 (−0.20 to 0.04)	0.19	−0.09 (−0.21 to 0.03)	0.15
Model 3	−0.09 (−0.16 to −0.01)	0.10	−0.10 (−0.23 to 0.04)	0.52	−0.08 (−0.20 to 0.05)	0.58	−0.08 (−0.20 to 0.05)	0.42
**AHI in REM sleep**								
Model 1	−0.06 (−0.14 to 0.02)	0.15	−0.08 (−0.22 to 0.06)	0.25	−0.11 (−0.23 to 0.02)	0.10	0.02 (−0.13 to 0.17)	0.79
Model 2	−0.06 (−0.13 to 0.02)	0.13	−0.08 (−0.21 to 0.05)	0.30	−0.08 (−0.20 to 0.04)	0.19	0.01 (−0.12 to 0.15)	0.84
Model 3	−0.05 (−0.12 to 0.03)	0.39	−0.07 (−0.20 to 0.07)	0.63	−0.07 (−0.20 to 0.06)	0.60	0.04 (−0.10 to 0.18)	0.68
**Mean oxygen saturation<94%**								
Model 1	−0.12 (−0.28 to 0.04)	0.14	0.07 (−0.19 to 0.32)	0.61	−0.35 (−0.64 to −0.06)	0.02	−0.10 (−0.41 to 0.22)	0.55
Model 2	0.01 (-0.14 to 0.16)	0.89	0.15 (−0.08 to 0.38)	0.31	−0.18 (−0.45 to 0.10)	0.20	0.01 (−0.27 to 0.30)	0.93
Model 3	0.02 (−0.13 to 0.18)	0.84	0.16 (−0.08 to 0.40)	0.56	−0.18 (−0.47 to 0.10)	0.44	0.03 (−0.27 to 0.33)	0.87

Data is presented as standardized β (95% confidence interval) with FDR-adjusted *p* value
Model 1 unadjusted. Model 2: adjust for age, sex, and race/ethnicity (in the model for overall sample). Model 3: model 2 plus further adjustment for body mass index, education, cognitive status, depressive status (history of depression or on anti-depressant), smoking, alcohol consumption, time interval between WatchPAT and brain MRI, and MRI scanner
AHI, apnea-hypopnea index; CI, confidence interval; FDR, false discovery rate; NHW, Non-Hispanic White; REM, rapid-eye movement

Table S3. Interaction *p*-values for sleep metrics × race/ethnicity on WMH volume

	Hispanic	Black	
	odds ratio (95% CI)	odds ratio (95% CI)	*p* value for interaction
**Sleep architecture**			
Light sleep percentage	0.99 (0.72 to 1.36)	1.06 (0.76 to 1.47)	0.91
Deep sleep percentage	0.97 (0.70 to 1.35)	0.92 (0.66 to 1.28)	0.88
REM sleep latency	0.78 (0.57 to 1.05)	0.74 (0.53 to 1.04)	0.22
REM sleep percentage	1.04 (0.76 to 1.41)	0.98 (0.70 to 1.36)	0.95
**Nocturnal hypoxemia**			
AHI	0.83 (0.59 to 1.17)	0.96 (0.59 to 1.17)	0.54
AHI in non-REM sleep	0.96 (0.69 to 1.33)	0.85 (0.61 to 1.19)	0.60
AHI in REM sleep	1.01 (0.73 to 1.40)	0.86 (0.61 to 1.21)	0.58
Mean oxygen saturation<94%	0.90 (0.46 to 1.74)	0.60 (0.31 to 1.16)	0.41

WMH volume was categorized into tertiles: the top tertile of WMH (largest WMH burden) to the lowest tertile of WMH (smallest WMH burden), using the lowest tertile as the reference group
Data is presented as odds ratio (95% confidence interval) with FDR-adjusted *p* value for interaction
Multivariable model used Non-Hispanic White group as the reference group
AHI, apnea-hypopnea index; CI, confidence interval; REM, rapid-eye movement

Table S4. Linear regression models investigating associations between sleep macro-architecture and hippocampal volume by race/ethnicity

	Overall (n = 777)		NHW (n = 325)		Hispanic (n = 261)		Black (n = 191)	
	β (95% CI)	p value	β (95% CI)	p value	β (95% CI)	p value	β (95% CI)	p value
**Light sleep percentage**								
Model 1	−0.12 (−0.19 to −0.05)	<0.001	−0.08 (−0.20 to 0.04)	0.19	−0.20 (−0.33 to −0.08)	0.001	−0.06 (−0.18 to 0.07)	0.36
Model 2	−0.01 (−0.08 to 0.05)	0.68	0.04 (−0.07 to 0.14)	0.62	−0.09 (−0.20 to 0.03)	0.23	−0.01 (−0.13 to 0.11)	0.86
Model 3	0 (−0.07 to 0.06)	0.94	0.02 (−0.08 to 0.13)	0.84	−0.05 (−0.17 to 0.07)	0.74	0 (−0.13 to 0.12)	0.94
**Deep sleep percentage**								
Model 1	0.12 (0.05 to 0.19)	<0.001	0.06 (−0.07 to 0.19)	0.34	0.23 (0.11 to 0.35)	<0.001	0.07 (−0.04 to 0.19)	0.22
Model 2	0.03 (−0.04 to 0.09)	0.48	−0.06 (−0.17 to 0.05)	0.49	0.14 (0.03 to 0.26)	0.02	0.02 (−0.09 to 0.14)	0.87
Model 3	0.02 (−0.05 to 0.08)	0.77	−0.05 (−0.16 to 0.06)	0.60	0.11 (−0.01 to 0.23)	0.30	0.01 (−0.11 to 0.13)	0.93
**REM sleep latency**								
Model 1	−0.04 (−0.11 to 0.03)	0.30	−0.02 (−0.13 to 0.10)	0.77	−0.13 (−0.24 to −0.01)	0.03	0.06 (−0.08 to 0.19)	0.41
Model 2	−0.03 (−0.09 to 0.04)	0.49	−0.02 (−0.12 to 0.07)	0.64	−0.06 (−0.16 to 0.05)	0.43	0.03 (−0.10 to 0.15)	0.77
Model 3	−0.01 (−0.07 to 0.05)	0.79	−0.02 (−0.11 to 0.08)	0.86	−0.05 (−0.16 to 0.06)	0.70	0.02 (−0.11 to 0.15)	0.91
**REM sleep percentage**								
Model 1	0.08 (0 to 0.15)	0.04	0.07 (-0.05 to 0.19)	0.23	0.11 (−0.01 to 0.24)	0.08	0.02 (−0.11 to 0.15)	0.78
Model 2	0 (−0.07 to 0.06)	0.90	−0.01 (−0.11 to 0.09)	0.83	0 (−0.11 to 0.12)	0.98	0 (−0.13 to 0.12)	0.95
Model 3	−0.01 (−0.07 to 0.05)	0.90	0 (−0.09 to 0.10)	0.94	−0.03 (−0.15 to 0.09)	0.91	0 (−0.13 to 0.13)	0.96

Data is presented as standardized β (95% confidence interval) with FDR-adjusted p value
Model 1 unadjusted. Model 2: adjust for age, sex, and race/ethnicity (in the model for overall sample). Model 3: model 2 plus further adjustment for body mass index, education, cognitive status, depressive status (history of depression or on anti-depressant), smoking, alcohol consumption, time interval between WatchPAT and brain MRI, and MRI scanner
CI, confidence interval; FDR, false discovery rate; NHW, Non-Hispanic White; REM, rapid-eye movement

Table S5. Linear regression models investigating associations between nocturnal hypoxemia and hippocampus volume by race/ethnicity

	Overall (n = 777)		NHW (n = 325)		Hispanic (n = 261)		Black (n = 191)	
	β (95% CI)	*p* value	*β* (95% CI)	*p* value	*β* (95% CI)	*p* value	*β* (95% CI)	*p* value
**AHI**								
Model 1	−0.11 (−0.18 to −0.04)	0.001	−0.17 (−0.30 to −0.04)	0.01	−0.07 (−0.18 to 0.05)	0.27	−0.10 (−0.22 to 0.01)	0.07
Model 2	−0.08 (−0.14 to −0.02)	0.02	−0.09 (−0.20 to 0.02)	0.17	−0.04 (−0.15 to 0.06)	0.62	−0.11 (−0.21 to 0)	0.08
Model 3	−0.06 (−0.12 to 0.01)	0.22	−0.05 (−0.17 to 0.06)	0.51	−0.02 (−0.13 to 0.10)	0.89	−0.10 (−0.21 to 0.01)	0.43
**AHI in non-REM sleep**								
Model 1	−0.12 (−0.19 to −0.05)	<0.001	−0.16 (−0.29 to −0.03)	0.01	−0.08 (−0.20 to 0.04)	0.17	−0.11 (−0.22 to 0)	0.05
Model 2	−0.08 (−0.14 to −0.02)	0.02	−0.06 (−0.17 to 0.05)	0.43	−0.05 (−0.16 to 0.06)	0.52	−0.11 (−0.22 to −0.01)	0.06
Model 3	−0.06 (−0.12 to 0.01)	0.21	−0.03 (−0.14 to 0.09)	0.86	−0.03 (−0.14 to 0.09)	0.85	−0.10 (−0.22 to 0.01)	0.40
**AHI in REM sleep**								
Model 1	−0.08 (−0.15 to −0.01)	0.02	−0.15 (−0.27 to −0.02)	0.02	−0.03 (−0.15 to 0.09)	0.60	−0.08 (−0.20 to 0.04)	0.21
Model 2	−0.07 (−0.14 to −0.01)	0.03	−0.12 (−0.22 to −0.01)	0.05	−0.02 (−0.13 to 0.09)	0.96	−0.07 (−0.19 to 0.04)	0.30
Model 3	−0.05 (−0.12 to 0.02)	0.35	−0.10 (−0.21 to 0.01)	0.35	0.02 (−0.10 to 0.14)	0.86	−0.08 (−0.20 to 0.04)	0.48
**Mean oxygen saturation<94%**								
Model 1	−0.22 (-0.36 to −0.08)	0.002	−0.19 (−0.42 to 0.04)	0.11	−0.25 (−0.52 to 0.02)	0.07	−0.17 (−0.43 to 0.09)	0.20
Model 2	−0.08 (−0.21 to 0.05)	0.29	−0.06 (−0.26 to 0.14)	0.62	−0.10 (−0.34 to 0.15)	0.68	−0.11 (−0.36 to 0.14)	0.59
Model 3	−0.06 (−0.18 to 0.09)	0.48	−0.04 (−0.24 to 0.16)	0.87	−0.08 (−0.34 to 0.18)	0.81	−0.10 (−0.35 to 0.16)	0.90

Data is presented as standardized *β* (95% confidence interval) with FDR-adjusted *p* value
Model 1 unadjusted. Model 2: adjust for age, sex, and race/ethnicity (in the model for overall sample). Model 3: model 2 plus further adjustment for BMI, education, cognitive status, depressive status (history of depression or on anti-depressant), smoking, alcohol consumption, time interval between WatchPAT and brain MRI, and MRI scanner
AHI, apnea-hypopnea index; CI, confidence interval; FDR, false discovery rate; NHW, Non-Hispanic White; REM, rapid-eye movement

Table S6. Interaction *p*-values for sleep metrics × race/ethnicity on hippocampal volume

	**Hispanic**		**Black**	
	β (95% CI)	*p* value for interaction	β (95% CI)	*p* value for interaction
**Sleep architecture**				
Light sleep percentage	−0.05 (−0.20 to 0.10)	0.82	0.02 (−0.14 to 0.17)	0.93
Deep sleep percentage	0.14 (−0.02 to 0.29)	0.22	0.03 (−0.13 to 0.18)	0.84
REM sleep latency	−0.03 (−0.17 to 0.11)	0.86	0.03 (−0.13 to 0.19)	0.86
REM sleep percentage	−0.04 (−0.19 to 0.11)	0.94	−0.05 (−0.20 to 0.11)	0.75
**Nocturnal hypoxemia**				
AHI	0.07 (−0.08 to 0.22)	0.55	0.01 (−0.14 to 0.17)	0.93
AHI in non-REM sleep	0.04 (−0.11 to 0.19)	0.77	−0.02 (−0.17 to 0.13)	0.87
AHI in REM sleep	0.12 (−0.03 to 0.27)	0.28	0.06 (−0.10 to 0.21)	0.63
Mean oxygen saturation<94%	−0.02 (−0.33 to 0.29)	0.98	0 (−0.31 to 0.32)	0.98

Data is presented as standardized β (95% confidence interval) with FDR-adjusted *p* value for interaction
Multivariable model used Non-Hispanic White group as the reference group
AHI, apnea-hypopnea index; CI, confidence interval; FDR, false discovery rate; REM, rapid-eye movement

Figure S1. Distribution of WMH volume

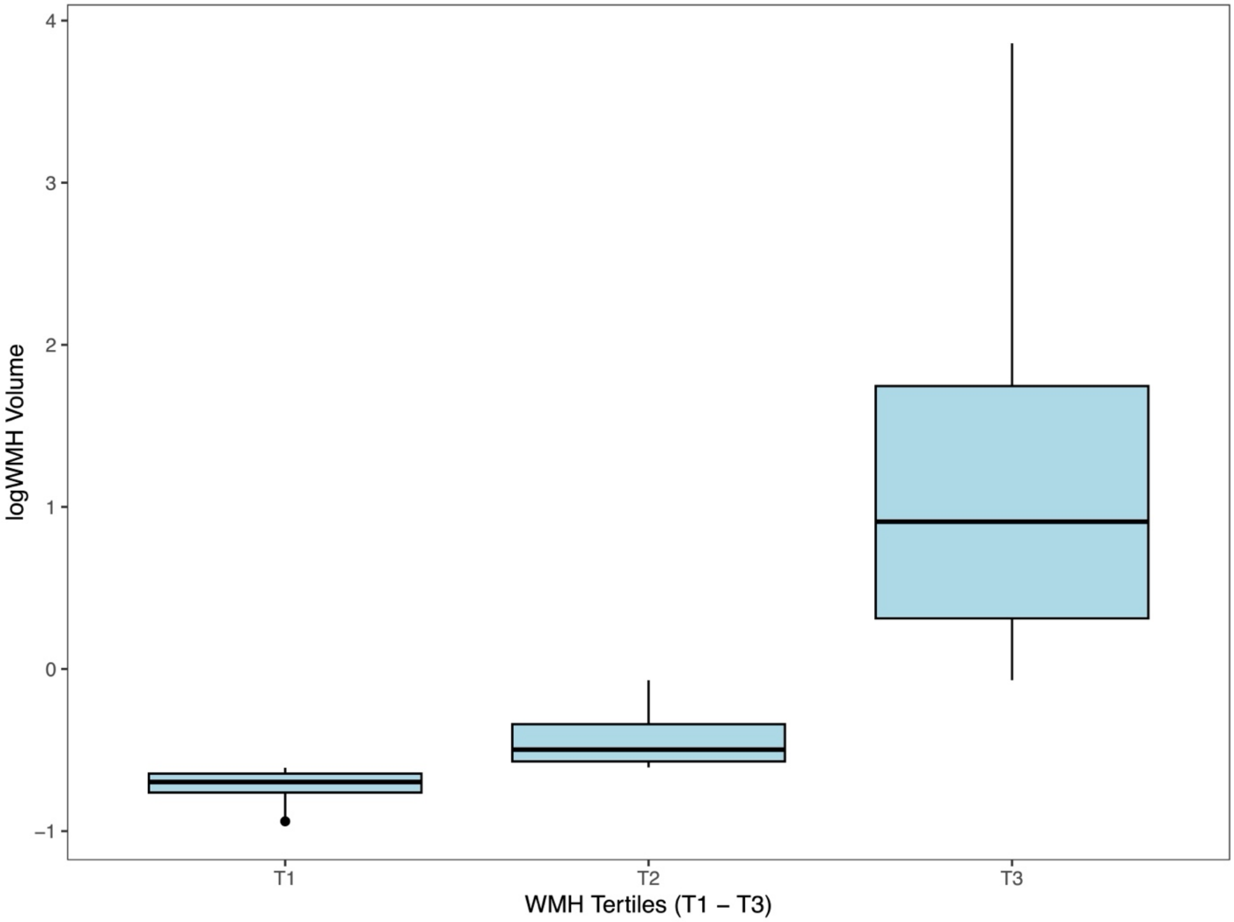

WMH, white matter hyperintensities

Figure S2. Odds ratios of the associations between sleep macro-architecture and WMH volume by race/ethnicity

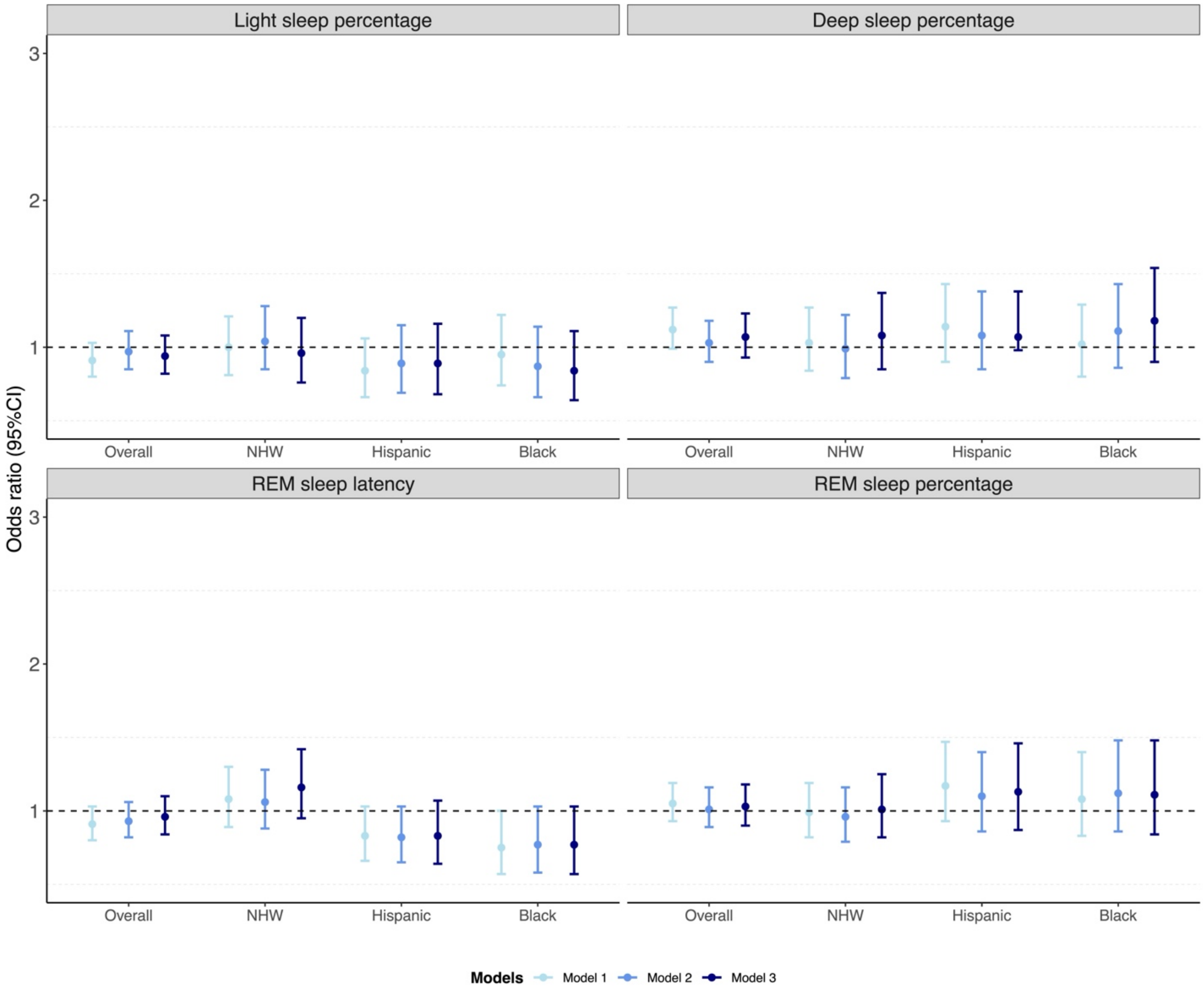

WMH volume was categorized into tertiles: the top tertile of WMH (largest WMH burden) to the lowest tertile of WMH (smallest WMH burden), using the lowest tertile as the reference group
Model 1 unadjusted. Model 2: adjust for age and sex. Model 3: model 2 plus further adjustment for education, body mass index, cognitive status, depressive status (history of depression or on anti-depressant), smoking, alcohol consumption, time interval between WatchPAT and brain MRI, and MRI scanner
*FDR-adjusted $p<0.05$; **FDR-adjusted $p<0.01$; ***FDR-adjusted $p<0.001$
CI, confidence interval; FDR, false discovery rate; NHW, Non-Hispanic White; REM, rapid-eye movement
